# Assessment of Serum Uric Acid, Urea, and Glucose Levels and Associated Factors among Breast Cancer Patients Attending A Tertiary Hospital in Bahirdar, Ethiopia: A Comparative Cross-Sectional Study

**DOI:** 10.4314/ejhs.v32i6.16

**Published:** 2022-11

**Authors:** Tinfash Kebede, Tadele Melak, Abu Ali Ibn Sina, Alebachew Fasil

**Affiliations:** 1 Department of Medical Laboratory science, Chief Clinical Chemist at Debark hospital, Debark, Ethiopia; 2 Department of Clinical Chemistry, School of Biomedical and Laboratory Sciences, College of Medicine and Health Sciences, University of Gondar, Gondar Ethiopia; 3 Center for Personalized Nanomedicine, Australian Institute for Bioengineering and Nanotechnology (AIBN), Corner College and Cooper Roads (Bldg 75), The University of Queensland, Brisbane, QLD 4072, Australia; 4 Department of Clinical Chemistry, School of Biomedical and Laboratory Sciences, College of Medicine and Health Sciences, University of Gondar, Gondar Ethiopia

**Keywords:** Breast cancer, Serum Uric acid, Urea, Glucose, Felege-Hiwot Hospital

## Abstract

**Background:**

Breast cancer is currently become a major public health problem in both developed and developing regions, it is one of the most common surgical problems in Ethiopia. Therefore, this study assessed serum uric acid, urea, and glucose levels and associated factors among benign, malignant breast cancer patients and apparently healthy women attending at Felege-Hiwot comprehensive Specialized Hospital.

**Methods:**

Hospital based comparative cross-sectional study was conducted among benign, malignant breast cancer patients and apparently healthy women attending at Felege-Hiwot Comprehensive Specialized Hospital. Out of 178 study participants 66 benign and 23 malignant fine needle aspirate cytology confirmed breast cancer patients and 89 apparently healthy women, included. Multivariable logistic regression models used to measure the strength of associations. A P value of < 0.05 was considered statistically significant.

**Results:**

Majority of the study participants, 81(91%) controls, 55(83.3%) benign, and 17(73.9%) malignant cases were premenopausal. Serum glucose 144.47±74.35 and uric acid 6.84±2.54 levels were significantly elevated in malignant cases than control (p-value< 0.05). Patients with malignant status were 4.38 times more likely to have hyperglycemia (AOR=4.38, 95%CI: 1.98–19.97) and 5.53 times more likely have hyperuricemia (AOR=20.43–95% CI: 6.80–61.23), 4 times more likely to have uremia (AOR=4.09, 95% CI: 1.06–15.91) compared to apparently healthy women.

**Conclusion:**

Serum glucose, and uric acid levels were significantly higher in malignant and benign cases compared with apparently healthy women. Family history of breast cancer, body mass index, systolic hypertension, comorbidity, residence and menopausal status were significantly associated with hyperglycemia, uremia and hyperuricemia.

## Introduction

Cancer is a group of diseases that cause cells in the body to change and grow out of control which forms a lump or mass called a tumor (1). Breast cancer (BC) begins in breast tissue which is made up of glands called lobules, and the ducts which connect lobules to the nipple. (2–4). Breast cancer is the leading cause of cancer death among females worldwide, with an estimated 2.3 million cases and 690,000 deaths in 2020. It accounts for 11.7% of all cancer cases and 6.9% of all cancer deaths among females (5).

In Ethiopia, BC the most prevalent cancer among the adult population, which accounts for 30.2% of the total cancer cases (6). BC like any other disease condition is associated with disturbing the body's physiological functions, change in homeostasis, and production of some biochemical metabolites (7).

Factors of BC depends on a woman's cumulative lifetime exposure to endogenous estrogen and her periodic use of exogenous hormones. Young age at menarche, delayed menopause, late first pregnancy and use of contraceptives increase the risk of BC (8). On the other hand, late menarche, early menopause, early first pregnancy, high parity, and prolonged lactation seem to have a protective effect (9). Blood chemistry is a common test used to evaluate different chemical components delivered from the body tissues. Measuring serum uric acid (SUA), urea and glucose can determine health and proper function of various organs like kidneys, liver, and other organs of BC patients (10).

Uric acid is the product of the metabolism of dietary or endogenous adenine and guanine-based purines, excreted by the kidney(11). A systemic antioxidant and its pro-oxidant properties of uric acid play an important role in the pathogenesis of cancer (13). Uric acid is an antioxidant only in the hydrophilic environment (14). Tumor-related factors include a high tumor cell proliferation rate, large tumor burden, and tumor chemo-sensitivity predisposing hyperuricemia (15). An excessive intracellular concentration of uric acid increased aggressiveness, and metastatic ability of breast cancer (16).

Urea is the primary metabolite derived from dietary protein and tissue protein turnover. Malignant tumor inappropriately metabolizes both dietary and host proteins, resulting in negative nitrogen balance. Breast cancer cells avoid the toxic effects of ammonia by converting the compound into usable nitrogen, a necessary agent for the rapid growth of tumors (17).

Glucose plays a role in the development of BC by favoring the selection of malignant cells (18). In addition, glucose may support carcinogenic processes through the generation of free radicals, and the induction of oxidative damage of both deoxyribonucleic acid (DNA) and the enzymes involved in the repair and processing of DNA (19)(20).

Elevated ROS promotes oxidation of DNA bases of guanine, resulting in “G” to “T” transversions. Oxidized bases lead to mutations that can trigger oncogenes or deactivate tumor suppressor genes, causing initiation and progression of multistage carcinogenesis (21)(22).

The levels of uric acid, urea, and glucose are mostly raised due to cancer cell metabolism or treatment response. Unless hyperglycemia, uremia, and hyperuricemia are diagnosed and managed early, they will lead to different comorbid diseases (23). If comorbidity is not diagnosed and managed properly, it will limit the BC treatment outcome, therefore this study tried to assess the level of uric acid, urea, and glucose levels among BC patients attending at Felege-Hiwot Comprehensive Specialized Hospital (FHCSH). We believe this analysis will significantly help clinicians with accurate diagnosing and therapy monitoring with better patient outcomes.

## Methods and Materials

**Study area, design, and period**: A hospital-based comparative cross-sectional study was conducted from February 01/2020 to April 30/2020 to assess serum uric acid, urea, and glucose levels and associated factors among benign breast lump, malignant breast tumor patients, and healthy women attending FHCSH. The study was conducted at FHCSH, a public hospital found in Bahir Dar city, Northwest Ethiopia. It serves around 7 million people in the catchment area.

**Study population**: All pathologically confirmed benign lump and malignant BC female patients with age ≥18 years, fulfilling inclusion criteria were study subjects. All healthy and BC-free women who visited the inpatient department of FHCSH during the study period were included as controls. Controls checked for BC, and chronic illnesses like renal and liver impairment; hypertension, DM, and pregnancy. All BC women patients having other cancer disease, women who were impractical for sample collection, and having known renal and liver impairment were excluded from this study.

**Sample size and sampling technique:** The sample size required for the study was calculated by comparing the mean difference of the two groups and by considering the following assumptions; confidence interval 95% and power 80%, the mean ± SD of UA was 3.723±0.911 and 4.104±0.901 for control and case, respectively which is taken from a study conducted in Libya(24). The final calculated sample size is 178, 89 cases, and 89 matched controls. A convenient sampling technique was used to recruit BC cases and matched controls at Felege-Hiwot comprehensive Specialized Hospital.

**Data collection and laboratory methods**

**Socio-demographic information**: Sociodemographic characteristics questions were collected using an interviewer-administered questionnaire after study subjects signed written informed consent. A semi-structured questionnaire prepared in the Amharic language and back translated to English and Amharic version was used for data collection.

**Anthropometric measurement**: Height and weight of study participants were measured with a stadiometer and digital weight scale (Zhongshan Frecom Electronic Company Limited, China) respectively to calculate BMI. The blood pressure of the study participants was measured by trained nurses with a manual aneroid sphygmomanometer manufactured by Shanghai Caremate medical Co., Ltd., Shanghai, China.

**Blood sample collection and processing**: Five-milliliter venous blood was collected using a serum separator tube labeled with the participant's identification number. All study participants were instructed to be restricted from taking food at least for 8 hours after interviewing and performing anthropometric measurements. Blood glucose, uric acid, and urea levels were determined using glucose oxidase, uricase enzymatic colorimetric method, and urease methods respectively using the Mindray BS-200E clinical Chemistry auto analyzer.

**Data management and quality control**: To ensure the quality of data, one-day training was given to data collectors by the principal investigator, and pre-test was done on 32(5%) of the total study population at Tibebe-Ghion Specialized hospital. The quality of results assured by running quality control samples (Humatrol P and Humatrol N) daily.

**Data analysis and interpretation**: Sociodemographic, clinical, and assay results were entered into the SPSS software version 23 package and the difference in variables were tested and analyzed. To summarize the characteristics of study participants, descriptive statistics were employed. Independent samples t-test was used to see the difference in the mean values of uric acid, urea, and glucose levels between cases and controls. Multivariable logistic regression was used to assess the association between the explanatory variables with hyperuricemia, hyperglycemia, and uremia. The Hosmer and Leeshawn goodness test were checked to assess the fitness of the model. The magnitude of the association was measured using the AOR and 95% CI. A *p*-value < 0.05 was considered statistically significant.

**The following operational definitions are used**

BMI: Underweight, <18.5 kg/m2 Normal weight, 18.5–24.9 kg/m2 overweight 25–29.9 kg/m2 and obesity≥30 kg/m2 (25). Hyperglycemia: is defined as a fasting blood glucose level above 130mg/dl (26). Hyperuricemia: is an elevated uric acid level in the blood, with serum uric acid level >6.0 mg/dL in adult (27). Uremia: is the condition of having high levels of urea in the blood >22mg/dl (28). Hypertension: DBP ≥90 mmHg or SBP ≥140 mmHg or both DBP ≥90 mmHg and SBP ≥140 (25).

**Ethical consideration**: The study was conducted after ethical approval is obtained from the research and ethical review committee of the School of Biomedical and Laboratory Science, College of Medicine and Health Sciences, University of Gondar. Ethics approval number for the study; Ref No. SBMLS/2463/2020 the study was conducted following the Declaration of Helsinki.

## Results

**Socio-demographic and clinical characteristics of study participants**: From a total of 178 study participants 66 benign and 23 malignant women breast cancer patients and 89 healthy women were taken as controls. Most of the study breast cancer patients were 18–28 years, married, and with no formal education; 55 (61.8%), 47 (52.8), and 34 (38.2%) respectively. From the 66 benign lumps, 47(71.2%) were benign fibroadenoma and 19(28.8%) were benign breast lesions, whereas among 23 malignant cases, 19(82.6%) of ductal carcinoma and 4(17.4%) of lobular carcinoma. (Supplementary Table 1).

**Reproductive characteristics of the study participants**: About 77(60.2%) of controls, 35(53.0%) of benign lumps, and 12(52.2%) of malignant cases were a user of contraceptives at least one time in their lifetime. Ten (11.3%) controls, 11(77.55%) benign, and 4(17.4%) malignant cases had a history of abortion. In all study groups, 81(91%) controls 55(83.3%) benign lumps, and 17(73.9%) malignant cases were found premenopausal women (Table 1).

**Table 1:** Behavioral and reproductive characteristics among apparently healthy women, benign and malignant BC patients attending Felege-Hiwot Comprehensive Specialized Hospital 2020

Variable	Category	Frequency (%)		

		Control(N=89)	Benign (N=66)	Malignant (N=23)
Contraceptive	Yes	55(61.2)	35(53.0)	12(52.2
use	No	34(38.8)	31(47.0)	11(47.8)
	Total	89	66	23
Type of	Injection	19(55.9)	19(54.3)	10(83.3)
contraceptive	Implanon	7(20.6)	11(31.4)	2(16.7)
	Pills	8(23.5)	5(14.3)	0(0)
	Total	34	35	12
History of	Yes	10(11.3)	11(77.55)	4(17.4)
abortion	No	79(88.7)	38(22.45)	17(73.9)
	Total	89	49	21
Age at	Early	2(2.2)	3(4.5)	1(4.3)
Menarche in	Normal	30(34.4)	18(27.3)	6(26.1)
year	Late	57(63.3)	45(68.2)	16(69.6)
	Total	89	66	23
Ministration	Regular	59(66)	44(66.7)	13(56.5)
cycle	irregular	30(34)	22(33.3)	10(43.5)
	Total	89	66	23
Menopausal	Premenopausal	81(91)	55(83.3)	17(73.9)
status	Postmenopausal	8(9)	11(16.7)	6(26.1)
	Total	89	66	23
Age of first	Early	35(77.7)	39(59)	20(87.0)
Pregnancy	Normal	10(22.3)	25(41)	3(13.0)
	Total	45	66	23

**Anthropometric and clinical measurements of the study participants**: Out of the 23 malignant and 66 benign case study participants, 4(6.1%) benign and 6(26.1%) malignant cases had systolic hypertension. Fourteen (21.2%) benign and 9(39.1%) malignant cases were overweight. In addition, 15(22.7%) benign and 8(38%) malignant cases had a first-degree family history of breast cancer. The majority of benign study participants 47(71.2%) were fibroadenoma, while 19(82.6%) malignant cases were ductal carcinoma and 4(17.4%) were lobular carcinoma (Table 2).

**Table 2:** Independent sample t-test among apparently healthy women, benign and malignant BC cases attending Felege-Hiwot Comprehensive Specialized hospital 2020

Biochemical	Women with Malignant BC (n=23) Mean ± SD	Control (n=89) Mean ± SD	P- value
Glucose (mg/dl)	144.47±74.35	98.13±18.03	≤0.011
Urea (mg/dl)	20.56±15.24	17.67±9.6	0.507
Uric acid (mg/dl)	6.84±2.53	5.46±1.91	≤0.011
	Women with Benign BC(n=66)	Control (n=89)	
	Mean ± SD	Mean ± SD	
Glucose (mg/dl)	135.23±29.53	98.13±18.03	0.011
Urea (mg/dl)	25.09±15.25	17.67±9.6	0.05
Uric acid (mg/dl)	6.45±1.99	5.46±1.91	≤0.01
	Women with Malignant BC(n=23)	Women with Benign BC(n=66)	
	Mean ± SD	Mean ± SD	
Glucose (mg/dl)	135.23±29.53	144.47±74.35	≤0.01
Urea (mg/dl)	25.09±15.25	20.56±15.24	0.18
Uric acid(mg/dl)	6.45±1.99	6.84±2.53	0.45

NB: SD= standard deviation, SUA= serum uric acid, P-value is calculated using independent t-test.

**Biochemical parameters**: Independent sample t-test showed that the mean level of serum glucose (135.23±29.53, P -value=0.011), urea (25.09±15.25, p-value= 0.05), and SUA (6.45±1.99, p-value=≤0.01) were found significantly higher in the Benign group as compared to the control group. The mean serum level of glucose (144.47±74.35, p-value=≤0.01) and uric acid (6.84±2.53p-value=0.018) were significantly higher in the malignant groups as compared to the control group (Table 2).

From 66 benign cases 6(9.1%), 30(45.5%), and 34(52.5%) were found hyperglycemia, uremic, and hyperuricemic respectively whereas from 23 malignant BC cases 11(47.8%), 9(39.1%) and 11(47.8%) were found hyperglycemic, uremic and hyperuricemic respectively (Table 3).

**Table 3:** Hyperglycemia, uremia, and hyperuricemia status among control, benign and malignant BC patients attending Felege-Hiwot Comprehensive Specialized Hospital 2020

Characteristics	Control (N=89)	Benign (N=66)	Malignant (N=23)	Total
Hyperglycemia	4(4.5%)	6(9.1%)	11(47.8%)	22
Uremia	10(11.2%)	30(45.5%)	9(39.1%)	49
Hyperuremia	14(15.7%)	34(52.5%)	11(47.8%)	59

**Factors Associated with hyperuricemia, hyperglycemia, and uremia**: In the bivariate analysis, BC status, age, unable to read and write educational status, overweight BMI, presence of comorbidity, ≥90 mmHg SBP, and history of abortion were found to be significantly associated with hyperglycemia. While in the multivariable analysis, history of abortion, ≥90 mmHg SBP, overweight BMI, and BC status were found independently associated with hyperglycemia (Table 4).

**Table 4:** Bivariable and multivariable analysis of glucose levels among apparently healthy, benign, and malignant breast cancer women attending Felege-Hiwot Comprehensive Specialized hospital 2020

Variable		Normal	Hyperglycemia	COR 95%CI	AOR95%CI	P-value
BMI	Normal	113	9	1.00	1.00	
	Underweight	22	3	1.71(0.43,6.83)		0.12
	Overweight	21	10	5.14(1.83,14.38)	5.14(1.89,33.88)	≤0.018*
Abortion	No	97	12	1.00	1.00	
	Yes	9	7	7.76(1.73,34.89)	6.28(1.98,19.97)	0.04*
SBP	Normal	146	15	1.00	1.00	
	Hyper	11	6	5.30(1.72,16.39)	5.30(1.58,43.9)	≤0.014*
Age	18–28	91	4	1.00	1.00	
category	29–39	60	13	4.93(1.53,15.84)		0.15
	≥40	6	4	15.17(3.02,76.14)		0.17
Educational status	Secondary school	49	3	1.00	1.00	
	Primary school	45	3	1.09(0.21–5.67)		0.36
	Unable RW	63	15	3.89(.6.1.07–14.19)		0.25
Menopausal	Premenopausal	144	13	1.00	1.00	
status	Postmenopausal	13	8	6.55(1.84,13.97)		0.18
Comorbidity	No	146	7	1.00		
	Yes	11	14	26.55(8.88,79.34)		0.15
	Control	89	0	1.00	1.00	
BC status	Benign	60	12	2.19(0.55,8.71	1.82(0.47,7.08)	≤0.019*
	Malignant	6	11	9.17(2.84,29.59)	4.38(1.09,17.50)	≤0.016*

AOR adjusted odds ratio, CI confidence interval, COR crude odds ratio, SBP systolic blood pressure, BMI body mass index

* significant association

Comorbidity, urban residence, postmenopausal status, overweight BMI, and late age at menarche were found to be significantly associated with uremia in bivariable analysis. However, in the multivariable logistic regression analysis urban residence (AOR=4.52, 95% CI: 1.31–15.57), the presence of comorbidity (AOR=2.70, 95% CI: 1.18–7.48) and postmenopausal status (AOR=2.89, 95% CI: 1.21–6.89) were independently associated with uremia (Table 5).

**Table 5:** Bivariable and multivariable analysis of urea level among apparently healthy women, benign and malignant breast cancer women attending Felege-Hiwot Comprehensive Specialized hospital 2020

Variable		Normal	Uremia	COR95% CI	AOR95%CI	P-value
Comorbidity	No	116	37	1	1.00	
	Yes	13	12	4.15(1.61,10.69)	2.70(1.18,7.48)	0.039
Residence	Rural	72	24	1.00	1.00	
	Urban	56	26	5.77(1.58,21.09)	4.52(1.31,15.57)	0.048
Menopausal status	Premenopausal	113	40	1.00	1.00	
	Postmenopausal	15	10	3.70(1.04,13.35)	2.89(1.21,6.89)	≤0.018
BMI	Normal	98	17	1.00	1.00	
	Underweight	24	8	2.12(0.91,4.89)		0.21
	Overweight	19	12	1.12(037,3.34)		0.31
Age at menarche	Normal	40	15	1.00	1.00	
	Early	2	4	5.33(0.88,32.20)		0.16
	Late	92	25	0.75(0.35,1.52)		0.17
	Control	89	0	1.00	1.00	
BC status	Benign	36	30	2.02(0.30–13.48)	1.09(0.43–2.82)	≤0.012
	Malignant	14	9	4.38(1.13,17.04)	4.09(1.06,15.91)	0.04

AOR adjusted odds ratio, CI confidence interval, COR crude odds ratio, BMI, body mass index, BC breast cancer p-value < 0.05= significant association

Regarding hyperuricemia, ≥90 mmHg SBP, FHBC, overweight BMI, presence of comorbidity, and BC status were found to be significantly associated with hyperuricemia in bivariable analysis. After performing multivariable analysis FHBC (AOR=3.38, 95% CI: 1.40–8.16), overweight BMI (AOR=4.59, 95% CI: 1.75–12.03), presence of comorbidity (AOR= 3.70, 95% CI: 1.42–9.39) and BC status (AOR= 20.43,95% CI: 6.82–61.23) significantly associated with hyperuricemia (Table 6).

**Table 6:** Bivariable and multivariable analysis of SUA among apparently healthy women, benign and malignant breast cancer women attending Felege-Hiwot Comprehensive Specialized hospital 2020

Variable	Category	Normal	Hyperuricemia	COR95%CI	AOR95%CI	p-value
SBP	Normal	120	41	1.00	1.00	
	Hyper	8	9	13.29(1.14,55.64)		0.32
FHBC	No	115	36	1.00	1.00	
	Yes	13	14	5.36(2.29,12.52)	3.38(1.40,8.16)	≤0.013
BMI	Normal	95	27	1.00	1.00	
	Underweight	16	9	0.26(0.07,0.94)	0.28(0.08,1.03)	0.02
	Overweight ≥25	17	17	6.04(1.3,27.94)	4.59(1.75,12.03)	≤0.011
Comorbidity	No	114	14	1.00	1.00	
	Yes	11	39	4.55(1.55,13.39)	3.70(1.42,9.39)	0.03
	Control	89	0	1.00	1.00	
BC status	Benign	36	30	0.16(0.07–0.37)	0.14(0.06–0.34)	≤0.01
	Malignant	11	12	7.18(1.17,43.96)	5.53(1.03–29.63)	0.04

AOR adjusted odds ratio, CI confidence interval, COR crude odds ratio, BMI, body mass index, BC breast cancer, FHBC family history of breast cancer, SBP systolic blood pressure, p-value < 0.05= significant association

## Discussion

In breast cancer patients' evaluation of biochemical levels is highly important in developing an effective strategy to prevent and treat comorbidities. Factors associated with BC, SBP, family history of breast cancer, comorbidity, residence, abortion, BMI, and menopausal status have been incriminated along with SUA, glucose, and urea level. Increasing the level of those chemicals in breast cancer patients can contribute to the development of other diseases (29,30).

The present study results demonstrated that there was significantly higher serum glucose (p-value=0.011), SUA (p-value ≤ 0.01), and urea (p-value=0.05) levels in a benign group compared with the control group. The level of glucose (p-value ≤0.01) and uric acid (p-value ≤0.01) were also significantly higher in malignant cases as compared with controls. Higher level of SUA in BC patients (p-value<0.05) compared with control are similar to a comparative cross-sectional study done at Tikur-Anbessa Specialized Teaching Hospital, Ethiopia (31).

In our study SUA was found higher in malignant BC patients compared with controls, (p-value ≤0.011), similarly study from Libya and Iraq reported an elevated level of SUA in benign breast tumors compared with controls (p-value = 0.0270 (24)(32). The reason for this elevated level of SUA in breast cancer patients may be due to increased massive destruction of surrounding tissues, which leads to nucleic acid turnover and consequent increase in the catabolism of purine bases in breast tumors (16).

In this study, serum urea level was significantly higher in a benign group compared with the control group (p-value=0.05). Serum urea level was nonsignificantly increased in malignant BC patients than in BC-free women. This finding is similar with the study conducted in India which showed that the level of urea was significantly increased in BC cases compared with controls (33).

In our study, BC patients exhibited higher levels of glucose in their serum compared to the control group. Studies done in Pakistan showed that serum glucose level was significantly raised in breast cancer cases as compared to control groups (30). The reason for this elevated level of serum glucose in breast cancer patients may be due to cancer cells actively producing more glucose transporters on their cell surface membranes, and more glucose is brought inside the cell. Glucose is broken down by aerobic glycolysis for ATP production speedily in the cell. Cancer cells exhibit aerobic glycolysis to get enough energy from glycolysis (34).

According to our findings, comorbidity among breast cancer patients were 13.6% DM and 8.7% systolic hypertension. The reason for DM and hypertension may be because of the effect of cancer cell metabolism. This finding is not similar to a study carried out in South Africa, which showed that 81.2% of the participants were obese, 61.3% were hypertensive and 47.1% were hyperglycemic. Study design and population variation may be possible reasons for the difference. Of all study participants, 91% of them had at least one metabolic disease (35).

In this study hyperuricemia is significantly associated with, comorbidity. The study done in India showed that comorbidities with BC were hypertension 21.8% and DM 16.7% was associated with hyperuricemia (36). The result was similar to our findings which are 13.6% DM and 8.7% systolic hypertension. On the other hand, patients with malignant tumors were 5.53 times more likely to have hyperuricemia than a benign lump. The reason may be; tumor lysis syndrome which leads to cell lysis, the release of purines, and a subsequent increase in uric acid level (16). Breast cancer patients who had FHBC were 3.38 times more likely to have hyperuricemia as compared to those without FHBC patients. Similarly, BC patients with comorbidity were 3.7 times more likely to have hyperuricemia than paints without comorbidity. In addition, overweight and obese patients are 4.59 times more likely to have hyperuricemia than normal-weight patients

In our study, patients who had malignant tumors were 4.38 times more likely to have hyperglycemia than a benign tumor. Possible explanations may be due to glucose being required to meet the metabolic demands of the fast proliferation of cancer cells and the increased rate of glycolysis and glucose transportation in malignant tissue (37). Breast cancer patients who had a history of abortion were 6.28 times more likely to have hyperglycemia compared to patients who had no history of abortion. In addition, patients with SBP were 5.30 times more likely to have hyperglycemia as compared to non-hypertensive participants. Breast cancer patients who were overweight and obese were 5.14 times more likely to have hyperglycemia than participants with normal weight. A similar study done in Spain showed that overweight BMI ≥ 25 BC patients had a significantly higher level of glucose compared with the control groups (38). Another similar study done in India showed that fasting blood glucose increase significantly with increasing BMI (p-value=≤ 0.011,). There was a significant positive correlation between BMI and fasting blood glucose *P*-value=≤0.011) (39).

In this study patients who had malignant tumors were 4.09 times more likely to have uremia than a benign tumor. Increased migration and invasion of breast carcinoma cells are key events in the development of metastasis to the lymph nodes and distant organs (40) which will induce an increased level of urea in the blood. This study result showed that urban resident BC patients were 4.52 times more likely to be uremic than rural participants. Possible reasons can be the nutritional difference, living status variation, and health-seeking behavior differences between urban and rural participants. Postmenopausal BC patients have 2.89 times more uremic than premenopausal patients. It is known that the relative risk of breast cancer increased with age at natural menopause (41), therefore postmenopausal may contribute to uremia induced by breast cancer. Breast Cancer patients who had comorbidity were 2.70 times more likely uremic as compared to patients without comorbidity.

In conclusion, our findings showed that serum glucose and uric acid levels were significantly higher in malignant and benign cases compared with healthy women. In benign cases, 9.1%, 45.5%, and 52.5% found hyperglycemic, uremic, and hyperuricemic respectively, whereas in malignant cases 47.8%, 39.1%, and 47.8% were hyperglycemia, uremic and hyperuricemic respectively. Patients who had FHBC, BMI ≥ 25, BC status, and systolic hypertension were significantly associated with SUA levels. Comorbidity, BC status, residence, and menopausal status were positive associations with uremia. History of abortion, SBP, BMI ≥ 25, and BC status were found independently associated with hyperglycemia.

Close monitoring of glucose, urea, and uric acid level should be done before and during treatment. Special focus should be given to malignant cases to prevent the development of comorbidity related to hyperglycemia, uremia, and hyperuricemia as well as to get early treatment before metastasizing. Further studies with a large sample size and follow-up study design are recommended.

The strength of the study was using standard test methods for SUA, urea, and glucose levels to assess the status of breast cancer patients at Felege-Hiwot Comprehensive Specialized hospital. Moreover, the inclusion of BC-free women as controls is another strength of the study. Assessing only three biochemical tests (blood glucose, urea, and SUA) is the first limitation of our study. Another limitation of the study is the inability to identify the level of SUA, urea, and glucose in different stages of breast cancer.
